# A proposal of an echocardiography-based pathway for the diagnosis and management of angina with no obstructive coronary arteries: a state-of-the-art review: Advancing Targeted Therapy and Prognosis Through Endotype Identification

**DOI:** 10.1093/ehjimp/qyag129

**Published:** 2026-07-22

**Authors:** Attila Kardos, Benjamin Bussmann, Maria George, Giovanni Luigi De Maria, Nicola Gaibazzi

**Affiliations:** Department of Cardiology, Translational Cardiovascular Research Group, Milton Keynes University Hospital NHS Foundation Trust, 8H Standing Way, Eaglestone, Milton Keynes MK6 5LD, UK; Faculty of Medicine and Health Sciences, University of Buckingham, Buckingham, UK; Department of Cardiology, Oxford University Hospital, Oxford, UK; Department of Physiology Anatomy and Genetics, University of Oxford, Oxford, UK; INOCA International, Manchester, UK; Department of Cardiology, Oxford University Hospital, Oxford, UK; AMIIC Catheterization Laboratory, University of Oxford, Oxford Heart Centre—Oxford University Hospital, Oxford, UK; Cardiology Department, Parma University Hospital, Parma, Italy

**Keywords:** ANOCA, INOCA, diagnostics, endotype, non-invasive pathway

## Abstract

In this paper we are discussing the currently available tools in the diagnosis of angina with non-occlusive coronary artery disease (ANOCA) a potentially disabling condition that affects approximately 30–60% of patients with angina. With the advancement in ultrasound technology, multi-parametric stress echocardiography in combination with hyperventilation provocation echocardiography lends itself as a bedside, scalable, safe and cost-effective test to triage patients who may require downstream invasive functional coronary angiography. The review is providing with the critical appraisal of the current invasive and non-invasive imaging modalities before the proposal of a fully non-invasive echocardiography-based pathway to identify ANOCA endotypes to allow delivery of targeted therapy for symptom control to avoid recurrent hospital admissions and to monitor treatment effect to improve quality of life and prognosis in ANOCA patients.

## Graphical Abstract

**Graphical Abstract:**
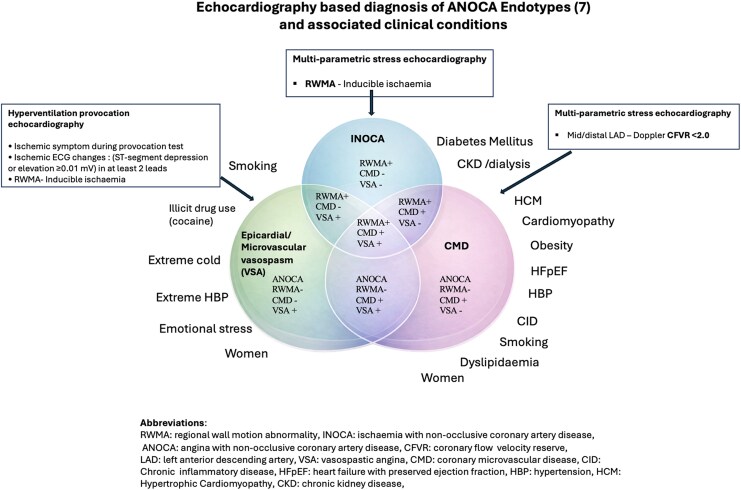

## Introduction

Chest pain is a common reason for primary care visits worldwide, accounting for about 1–2% of all consultations,^1–3^ approximately 5.6% of all emergency department visits in the US per year^4^ and 0.5% of outpatient clinic visits.^5^ Chest pain can be divided into cardiac and non-cardiac causes. Angina is the most common cause of cardiac chest pain, resulting from myocardial ischaemia secondary to coronary artery disease (CAD). However, about 50% of patients referred to coronary angiography for evaluation of angina have unobstructed epicardial coronary arteries, a condition called angina with non-occlusive coronary arteries (ANOCA). Of these up to 75% have evidence of coronary microvascular dysfunction (CMD) as the cause of myocardial ischaemia.^6,7^

The coronary arterial system is composed of large epicardial arteries which function primarily as conductive vessels, and intramyocardial coronary arterioles (IMCA) which function as the resistive vessels responsible for regulating blood flow to the heart.^8,9^ In CMD, the regulation of blood flow by IMCAs is impaired, leading to myocardial ischaemia.^8^ The mechanisms underlying CMD are heterogenous, including structural and functional alterations of IMCAs. Functional alterations such as endothelial and vascular smooth muscle dysfunction, can result in impaired vasodilatation or enhanced vasoconstriction. Structural abnormalities include diffuse or focal atherosclerosis, intimal thickening, smooth muscle cell thickening or proliferation, perivascular fibrosis/inflammation, capillary obstruction or reduced capillary diameter or density (rarefaction), as well as microembolization after atherosclerotic plaque rupture or during coronary intervention.^7,8,10^ CMD can thus be considered an umbrella term for a heterogenous group of conditions that ultimately lead to myocardial ischaemia in the absence of epicardial coronary stenosis.

In clinical practice the diagnosis of ANOCA can be challenging due to the heterogenous nature of its underling pathophysiology. The gold standard for the diagnosis of CMD is invasive functional coronary angiography (IFCA) which involves passing small sensory tipped wires into the coronary arteries.^11–13^ Coronary artery blood flow is then measured at rest and in response to provocative agents such as adenosine and acetylcholine. Changes in blood flow induced by these provocative agents can then be used to calculate several indices microvascular function, such as coronary flow reserve (CFR) and index of microvascular resistance (IMR) as well as detect the presence of coronary artery vasospasm.

**Figure 1:**
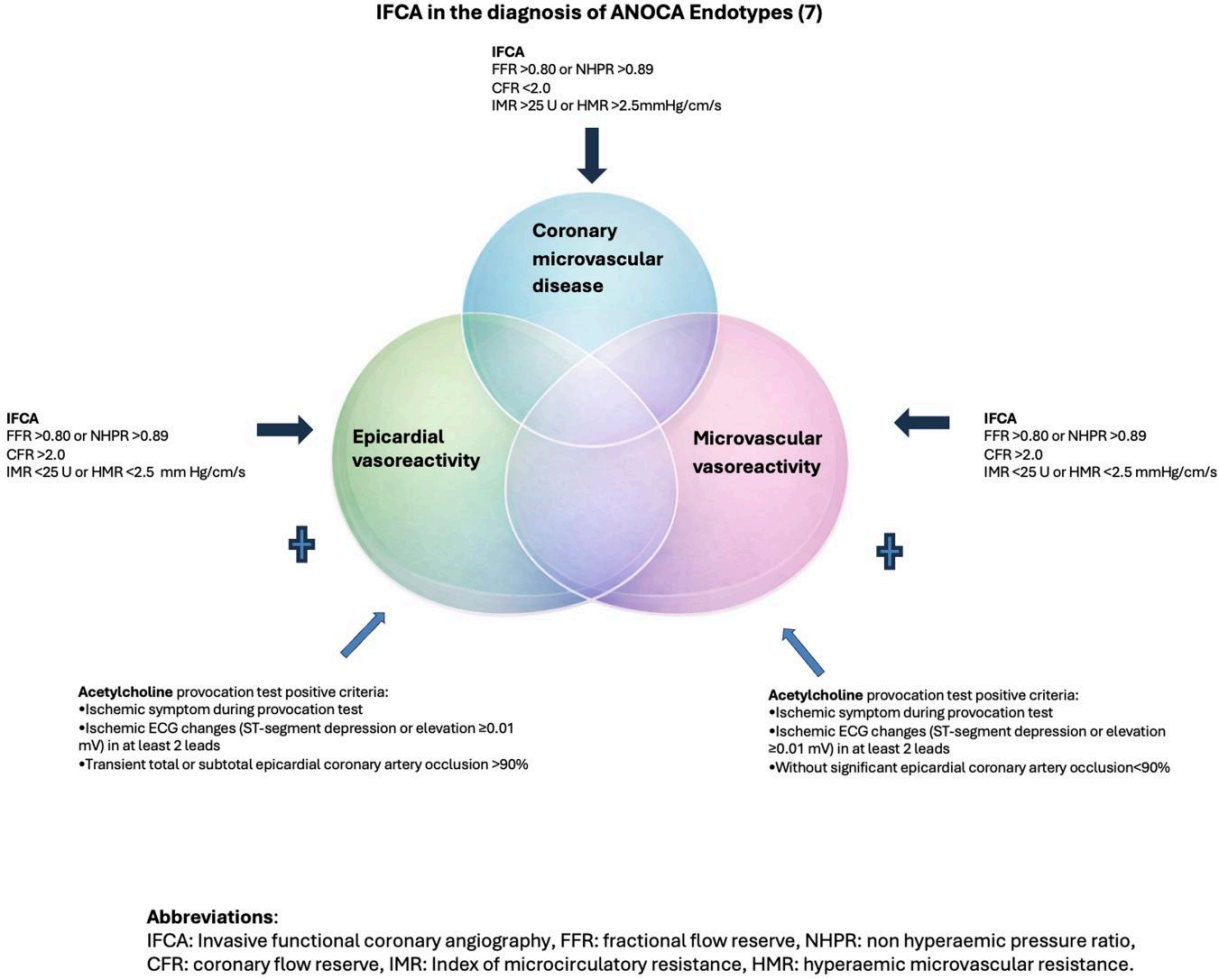
IFCA in the diagnosis of 7 ANOCA endotypes. IFCA, invasive functional coronary angiography; FFR, fractional flow reserve; NHPR, non-hyperaemic pressure ratio; CFR, coronary flow reserve; IMR, index of microcirculatory resistance; HMR, hyperaemic microvascular resistance.

Based on these indices of microvascular function, 3 different endotypes of ANOCA have been proposed; Coronary microvascular disease, Epicardial vasospastic angina, Microvascular vasospastic angina. Furthermore, in many patients more than one endotype may exist resulting in overlapping endotypes as a combination of 2 or all 3 of the individual endotypes (*Figure 1*).

To avoid the risks inherent in IFCA, non-invasive functional tests have been developed to assess indirectly the effect of the structural and functional abnormalities of the coronary microcirculation and is recommended by the current guidelines at IIa and IIb level to assess suspected ANOCA patients.^6,14^ Positron Emission Tomography (PET), Cardiac Magnetic Resonance imaging (CMR) are assessing in a semi-quantitative and in a quantitative way the myocardial or coronary flow reserve during maximal vasodilatation as a ratio of the maximal over the resting flow (CFR/MBFR). Doppler echocardiography-based coronary flow velocity reserve (CFVR) in the mi/distal left anterior descending artery (LAD) during stress echocardiogram has been shown to be feasible for both diagnostic and prognostic purpose.^15,16^ Neither of the non-invasive tests can address macro or microvascular vasoreactivity and hence, provide a comprehensive diagnostic for patients with ANOCA. In addition, IFCA for the assessment of vasoreactivity uses ‘pharmacological dose’ of intracoronary acetylcholine raising queries over its safety and diagnostic accuracy.^17^ ANOCA is associated with high rates of morbidity, including an impaired quality of life and recurrent hospitalizations, resulting in significant utilization of health care resources.^18–21^ The need to develop a comprehensive non-invasive, scalable diagnostic pathway for all the currently identified ANOCA endotypes is emerging.

### Non-invasive diagnostic tests for ANOCA

#### Positron emitting tomography in the diagnosis of ANOCA

**Figure 2:**
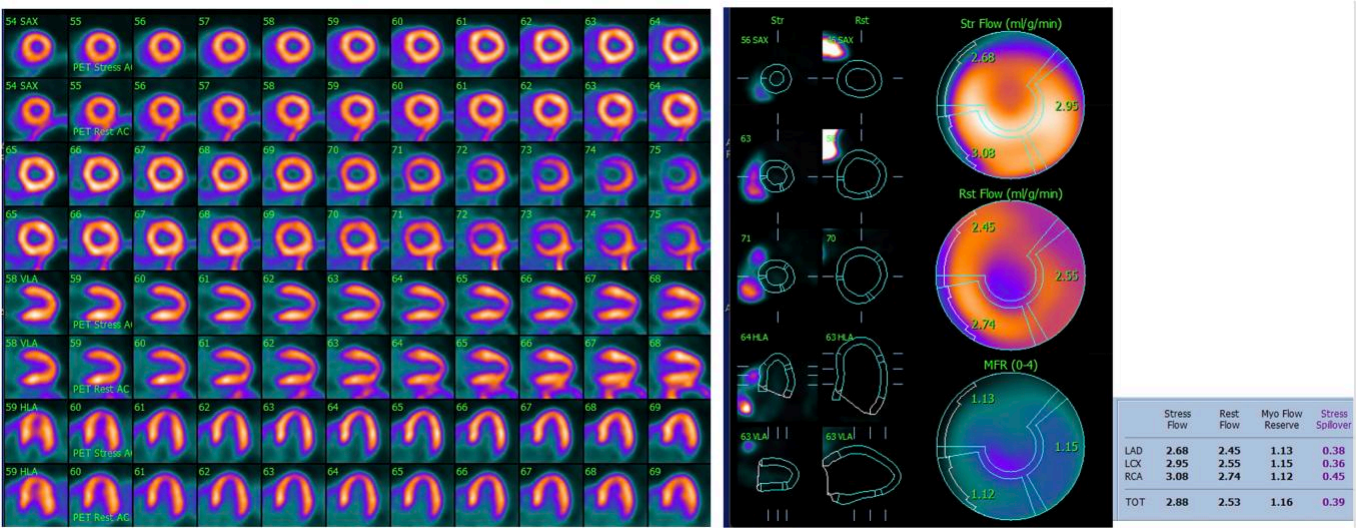
Case of a 71-year-old female with history of effort chest pain and breathlessness. An invasive angiogram confirmed normal coronary arteries. Regadenoson stress Rb82 PET perfusion scintigraphy showed no-inducible ischaemia (*A*) but reduced myocardial flow reserve (*B*) in all the three coronary artery territories in keeping with CMD.

PET plays a crucial role as a non-invasive modality in the diagnosis of ANOCA. PET enables the absolute quantification of myocardial blood flow (MBF), providing essential insights into coronary microvascular function. By measuring MBF both at rest and under stress conditions to quantify MBF-reserve, PET has high accuracy and reproducibility to identify impaired coronary vasodilator capacity, thus aiding in the diagnosis and risk stratification of patients with suspected CMD.^22^ PET radiotracers differ in availability, image quality, extraction fraction and MBF accuracy due to extraction linearity at high flow rates. Rubidium-82 (82Rb), nitrogen-13 ammonia (13N-NH_3_), oxygen-15 water (15O-H_2_O) are the most common tracers most of which require cyclotron except for rubidium^23^ making easy access difficult for PET (*Figure 2* and *Table 1*).

**Table 1:** Comparison of invasive and non-invasive CFVR/MBFR measurement techniques

	Invasive FCA CFR/FFR/IMR	Doppler –ECHO CFRV	PET MBF/MBFR	CMR MPR/MBFR
**Accuracy**	**++++**	**++**	**++++**	**+++**
**Reproducibility**	**++++**	**+++**	**++++**	**+++**
**Prognostic validation**	**+++**	**+++**	**++++**	**++**
**Complete ANOCA endotype assessment**	Yes	NO (Yes, with the proposed HPE, vasoreactivity assessment)	NO	NO
**Availability**	+++	++++	++	++
**Cost**	££££	£	£££	£££
				
**Radiation exposure**	Yes	No	Yes	No
**Invasive method with adherent SEA**	Yes	No	No	No
**Technical considerations**	Intra-arterial catheter technique with several pressure and temperature wire manipulation in the coronary arteries with inherent complications including MI, stroke and death. Pharmacological agent administration intra venously to assess coronary physiology at rest and during maximal vasodilatation.	Broad band transducer with second harmonics capability and low MI image acquisition for ultrasonic contrast images. CFVR- feasibility: LAD (95%), RCA (70%), LCX (60%) Resting and stress images by pharmacological agents (dipyridamole or dobutamine or supine ergometer exercise)	Stress and rest perfusion imaging using vasodilator agents	Dynamic acquisition of first pass perfusion during vasodilator stress followed by resting imaging. Paramagnetic gadolinium iv contrast usage. Quantitative analysis requires dual bolus or dual sequence imaging to enhance measurement precision and MFR.
**Cut-off values of abnormality**	CFR < 2.3 IMR >25 U MMR<2.7	CFVR < 2	CFR < 2	MPRI < 2 (quantitative: MBF and MFR)
**Dependence on operator skills**	Yes	Yes	Yes	Yes
**Special technical requirements and other consideration**	Availability of catheter lab and invasive techniques for coronary physiology assessment	No—UEA improves Doppler flow detection. Capable of detecting variations in coronary flow velocity but not absolute flow volume.	Rubidium-82, O-15 water, N13-ammonia, isotope tracer with cyclotron. Excellent diagnostic performance. High-resolution images with short scan duration	Long examination protocol, variable patient compliance, need to exclude obstructive CAD on anatomical images, Patient with claustrophobia -not tolerating test (10% prevalence) Contraindicated for patients with severe CKD, on haemodialysis, non-MRI safe cardiac devices, Arrhythmias, Weight constraint in obese patients (160 kg). Gadolinium paramagnetic contrast agent
**Suitable for follow up to assess efficacy of treatment**	No	Yes	No (availability, radiation and cost implication)	No (availability and cost implications)

FCA, functional coronary angiography; PET, positron emitting tomography; CMR, cardiac magnetic resonance; CTP, perfusion computer tomography; LAD, left anterior descending; LCX, left circumflex; RCA, right coronary artery; CFVR, coronary flow reserve; MBFR, myocardial blood flow reserve; MPRI, myocardial perfusion reserve index; MFR, myocardial flow reserve; UECA, ultrasound enhancing contrast agent; HPE, hyperventilation provocation echocardiogram; MRR, microvascular resistance reserve.

#### Cardiac magnetic resonance imaging in the diagnosis of ANOCA

**Figure 3:**
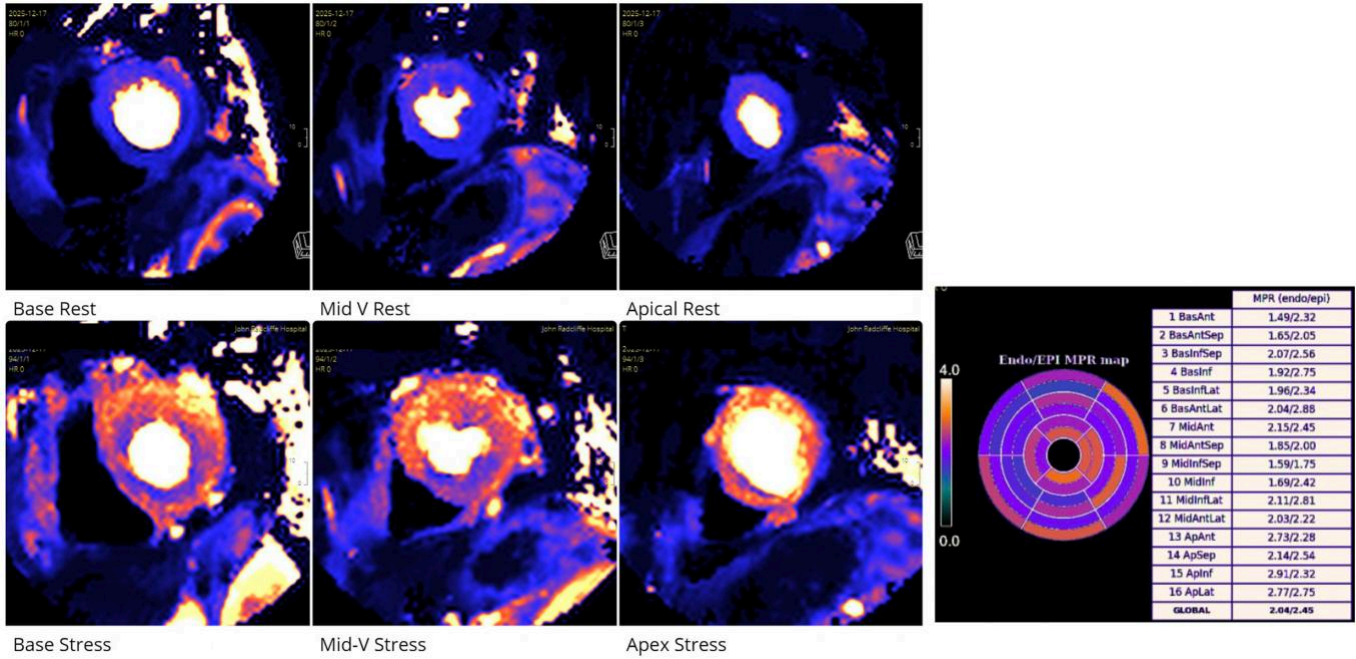
Case of a 65-year-old female with history of typical angina and effort breathlessness on the background of dyslipidaemia. Invasive coronary angiography showed mild coronary atherosclerosis in the LM, LAD and RCA. Regadenoson stress CMR perfusion images showed circumferential reduced endocardial perfusion in the base and mid segments with reduced myocardial flow reserve index (<2.0) in keeping with CMD.

CMR imaging has emerged as a pivotal non-invasive diagnostic tool in the evaluation of patients with ANOCA, particularly for the assessment of CMD. Utilizing advanced stress perfusion protocols, CMR enables the detailed characterization of myocardial perfusion abnormalities by comparing blood flow during pharmacologically induced vasodilator stress (commonly with adenosine) to resting conditions. This approach leverages semi-quantitative and fully quantitative analytical techniques, with improving levels of diagnostic precision.^24^ Quantitative CMR, in particular, allows for the measurement of MBF in absolute terms (mL/min/g), facilitating the detection of subtle microvascular impairments that may not be apparent using traditional methods^24–27^ Validation against gold-standard modalities such as PET and invasive coronary physiology testing underscores the robust diagnostic accuracy of CMR in this context. Consequently, CMR serves as an indispensable modality for elucidating the underlying microvascular pathology in ANOCA, guiding both prognosis and therapeutic decision-making in affected patients,^28–32^ (*Figure 3* and *Table 1*).

#### Doppler echocardiography in the diagnosis of ANOCA

**Figure 4:**
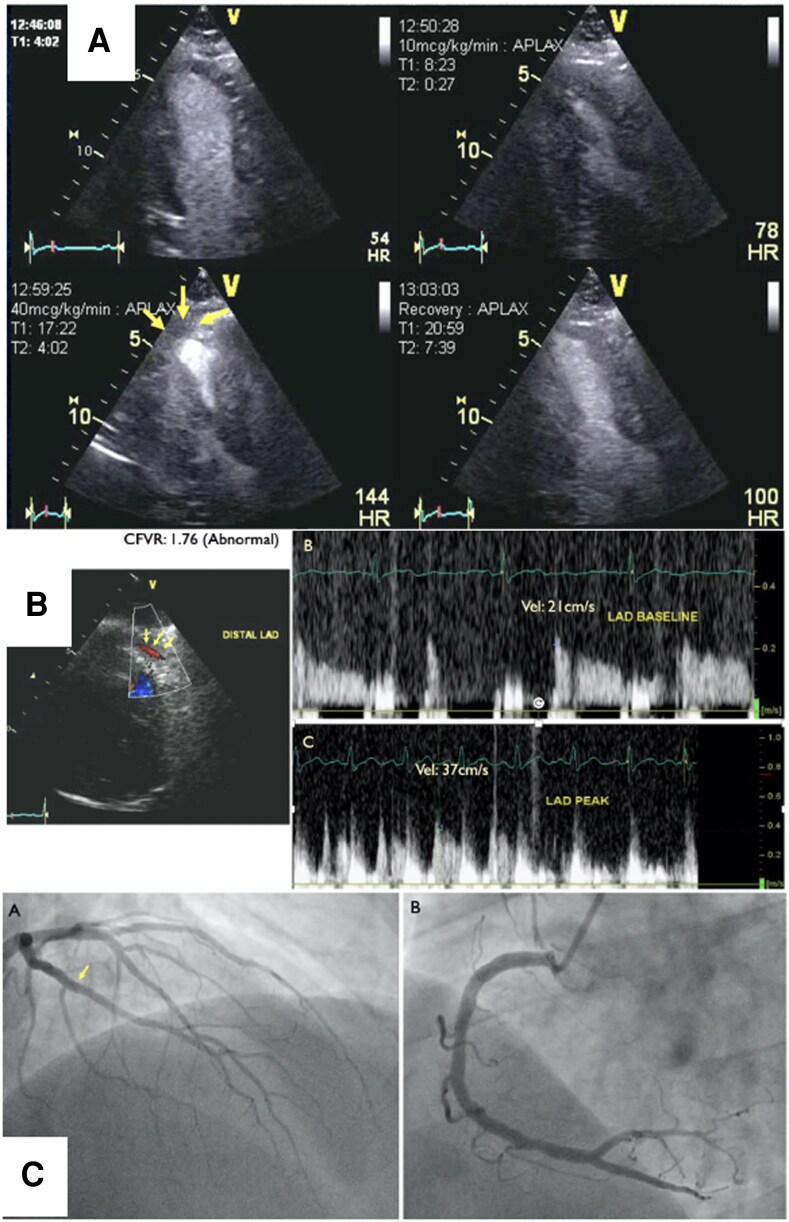
Case of a 53-year-old man with history of angina and prior PCI—for recurrence of typical angina. (*A*) Dobutamine Stress Echocardiography: Test was terminated at the end of protocol at 86% of target heart rate due to progressive angina. No resting RWMA, and all segments of the left ventricle augmented well during low dose. Peak stress: the apical segment became thin and dyskinetic (arrows). (*B*) The CFV in the distal LAD was 21 cm/s and 37 cm/s at baseline and peak, in keeping with reduced CFVR: 1.76. (*C*) Invasive coronary angiography confirmed non-occlusive CAD—diagnosis of ANOCA was made.

Doppler echocardiography of the LAD for CFVR measurement is increasingly recognized as an essential tool in the evaluation of patients with ANOCA.^33^ This non-invasive technique allows the assessment of microvascular function by comparing blood flow velocities in the mid/distal LAD at rest and during pharmacologic or exercise-induced stress test. A reduced CFVR, particularly values below 2.0, is indicative of CMD, a key underlying mechanism in ANOCA.^34,35^ Studies showed good correlation between thermodilution-derived CFR and CFV reserve (CFVR), while showing a stronger correlation with PET imaging.^36–40^ The method’s practicality, cost-effectiveness, and bedside applicability make it especially valuable in settings where more advanced imaging modalities such as PET or invasive assessments are not readily accessible. Nevertheless, its accuracy is dependent on operator expertise and image quality, and it is typically limited to the evaluation of a single coronary vessel. Despite these limitations, Doppler-derived LAD CFVR remains an essential component in the diagnostic algorithm for ANOCA, aiding in both risk stratification and guidance of further management (*Figure 4* and *Table 1*).

#### Invasive angiography—IFCA—to assess CMD

Invasive assessment of coronary microvascular system requires the introduction of sensory tipped wires into the coronary arteries. Pressure sensors allow measurement of pressure drops across arterial territories and thermal or Doppler sensors allow estimation coronary artery blood flow using thermodilution or Doppler velocity-based techniques.^11–13^ To assess coronary vasodilatory capacity blood flow is then measured at rest and during hyperaemia induced by adenosine infusion. Adenosine acts directly on smooth muscle cells through A2 receptors to induce hyperaemia through endothelium-independent vasodilation.

Functional flow reserve (FFR), calculated as the ratio of distal (P_d_) to proximal coronary (Pa) artery pressure during hyperaemia (FFR = P_d_/P_a_), is a measure of the haemodynamic significance of epicardial stenosis. Lesions with an FFR > 0.80 are considered non-flow limiting and have a favourable prognosis without intervention.^41,42^

The most widely used technique to estimate coronary blood flow is through bolus thermodilution which relies on the injection of cold saline boluses into the coronary arteries.^7^ The temperature change induced by the saline injection are measured by a thermal sensor in the coronary artery to produce thermodilution curves and calculate the transit time of the saline bolus. As coronary flow (Q) is inversely proportional to the transit time (T_transit_) of saline in the coronary artery (Q ∼1/T_transit_), T_transit_ can be used as a surrogate for coronary artery blood flow. Like this CFR can be calculated by comparing T_transit_ at rest and during hyperaemia (CFR = T_transit rest_/T_transit hyperaemia_). Similarly, based on ohms law, microvascular resistance can be estimated as the product of T_transit_ and distal coronary artery pressures (P_d_). IMR—an estimation of minimal microvascular resistance during hyperaemia is calculated during hyperaemia (IMR = T_transit hyperaemia_×P_d hyperaemia_).

While IMR and CFR are the current clinical standard for invasive assessment of the coronary microcirculation, they are imperfect. They rely on surrogate measures of coronary blood flow rather than coronary flow itself and suffer from significant inter-observer reliability due to practical challenges producing reproducible saline injections for thermodilution.^43,44^

Recently it has become possible to directly measure coronary blood flow through continuous thermodilution. This technique involves the controlled infusion of saline into the coronary arteries via a dedicated RayFlow infusion catheter using programmable injector pumps.^45^ Based on indicator-dilution theory real-time temperature changes induced by saline infusion are then used to calculate absolute volumetric coronary artery blood flow in mL/min^46^ and absolute microvascular resistance in wood units.^47^ These can then be used to calculate microvascular resistance reserve (MRR) a novel measure with is specific to microvascular function, independent of co-exiting epicardial disease.^48^ Continuous thermodilution measurement are highly reproducible, safe and are increasingly considered the gold standard for invasive measurement of coronary flow and microvascular resistance.^49^

In the absence of epicardial stenosis a CFR of <2.0–2.5 is the diagnostic hallmark of CMD and is an extensively validated marker of adverse long-term outcomes^50–52^ irrespective of underling IMR. An IMR of >25 is considered abnormal^11^ and is indicative of increased microvascular resistance but the independent prognostic significance of abnormal IMR in patient with ANOCA remains unclear.^53^ However, in the context of acute coronary syndrome significantly elevated IMR of >40 has been shown to predict complications, heart failure and mortality.^54–58^

The normal values derived from continuous thermodilution are less well defined. In normal subjects, LAD blood flow ranges form 50–100 mL/min at rest to 175–350 mL/min during hyperaemia.^59,60^ Microvascular resistance varies from 1200–1800 WU at rest to 200–470 WU during hyperaemia, with >500 WU considered abnormal.^43,60,61^ In a cohort of over 200 ANOCA patients an MRR of >2.7 excluded CMD^62^  *Table 1*. Nevertheless, a threshold of 3.0 has been shown to predict adverse long-term outcomes in large registry studies^63,64^ and response to antianginal therapy.^65^

#### Invasive technique to assess coronary artery vasoreactivity

Acetylcholine vasoreactivity testing is the current gold standard for assessing endothelial function. It has excellent safety record with no deaths and only 0.5% complication rate recorded in a meta-analysis of over 12 000 patients.^66^ Acetylcholine acting though endothelial muscarinic receptors induces release of vasodilatory factors such as nitric oxide (NO), to induce endothelium-dependant vasodilation. Acetylcholine also acts directly on smooth muscle cells where it induces vasoconstriction. In health the endothelial-dependant vasodilatory effects predominate, and intra-coronary injections of acetylcholine induce vasodilation and increase in coronary blood flow. However, in the presence of endothelial dysfunction or smooth muscle cell hypersensitivity, constriction predominates, leading to decreased coronary blood flow and even coronary vasospasm.^7,67^

Due to its short half-life acetylcholine must be administered intra-coronary via the coronary guiding catheter or through dedicated infusion catheters. The dosing protocols for acetylcholine vasoreactivity testing vary widely in clinical practice and medical literature with doses starting as low as 0.36 microgram to a maximum dose as high as 200microgram.^7,66^ In general, vasoreactivity testing consists of graded injections of increasing concentrations of acetylcholine. Lower doses assess endothelial function, triggering NO-mediated endothelial-dependant vasodilation in healthy endothelium or vasoconstriction in the presence of endothelial dysfunction. A failure to augment coronary artery blood flow by 50% in response to acetylcholine is considered abnormal and indicative of microvascular endothelial dysfunction and associated with poorer outcomes.^68,69^ Acetylcholine doses are then increased up to a maximum of 100–200 mcg to test for the presence of coronary vasospasm. The diagnostic criteria for vasospastic angina are set out by the Coronary Vasomotor Disorders International Study working group.^70^ Epicardial spasm is defined by a reduction in coronary diameter exceeding 90% accompanied by clinical symptoms and ischaemic ECG abnormalities during higher dose acetylcholine testing, while microvascular spasm occurs with chest pain and ECG changes indicative of ischaemia but with <90% luminal narrowing in the epicardial vessels. (*Figure 1*).

**Figure 5:**
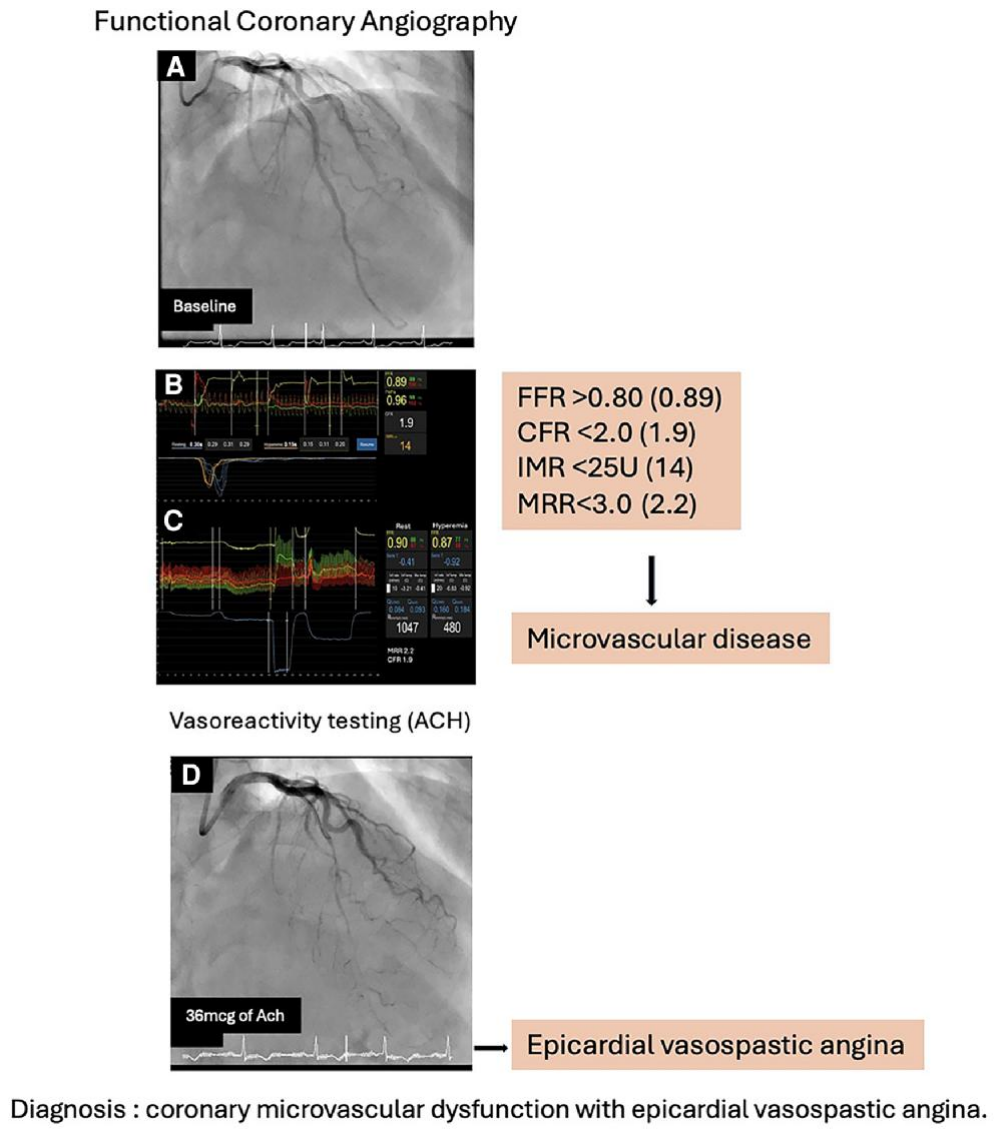
Case of a 60-year-old female patient with history of typical angina. CT coronary angiogram showed unobstructed coronary arteries, hence was referred for invasive microvascular assessment. (*A*) Baseline invasive coronary angiography confirmed unobstructive coronary arteries at baseline. (*B*) Bolus thermodilution microvascular assessment of the LAD showed normal fractional flow reserve (FFR) of 0.89 and index of microvascular resistance (IMR) of 14 and impaired coronary flow reserve (CFR) of 1.9. (*C*) Continuous thermodilution showed impaired microvascular resistance reserve (MRR) of 2.2 (*D*) On acetylcholine vasoreactivity testing, there was marked epicardial coronary spasm associated with a drop in distal coronary pressure which completely resolved after nitrate. Coronary microvascular dysfunction with epicardial vasospastic angina was diagnosed.

In the CorMicA trial, which looked at IFCA, 17% of participants demonstrated isolated vasospastic angina and approximately 21% showed overlapping features of microvascular and vasospastic angina, highlighting the limitations of current non-invasive tests that only able to assess CMD by measuring MFR or CFR but failing to detect micro and macro vascular vasoreactivity.^71^ By combining adenosine and acetylcholine testing, IFCA can assess both pathways of CMD, and vasoreactivity providing information of ANOCA endotypes. (*Figure 1* and *Figure 5*). CorMicA provides randomized control evidence that guiding treatment based on comprehensive IFCA improves patient outcomes^71^ and is cost effective.^72^ Indeed, acetylcholine provocation as part of a comprehensive coronary assessment is recommended in both UK and European consensus documents.^51^ It carries a class IIb recommendation for the assessment of patient with suspected coronary microvascular angina in the 2024 ESC guidelines,^6^ and a IIa recommendation for the assessment of patients with suspected ANOCA in the 2021 AHA guidelines for the evaluation and diagnosis of chest pain.^17^

However, the IFCA protocol often involves a pharmacological dose of acetylcholine injection into the coronary arteries selectively such as 2, 20, 100 µg/mL in 6 mL solution equating 12, 120, and 550 mg doses which is a non-physiological stimulus for vasospasm even in case of endothelial dysfunction. In addition, for safe acetylcholine injection in the right coronary artery to bail out sever bradycardia (AV block) a temporary pacemaker wire is also inserted. There is a need for a physiological validated safe test to be implemented for vasoreactivity testing that can complement CFVR derived CMD assessment to identify the ANOCA endotypes non-invasively *Table 1*.

### Proposal of a fully non-invasive echocardiography based ANOCA endo-typing

To overcome the limitations of non-invasive modalities in providing a comprehensive classification of ANOCA endotypes and to avoid invasive coronary physiological tests with its inherent risk of complications and cost implications we are proposing a fully non-invasive pathway based on echocardiography. Using Coronary Computed Tomography Angiography (CCTA) as an anatomical test to rule out occlusive CAD (no or <50% diameter stenosis) in patients with typical chest pain or breathlessness we propose *multi-parametric stress echocardiography* (MPSE) to assess inducible ischaemia (regional wall thickening), mid/distal LAD CFVR to assess CMD, in combination with *hyperventilation provocation echocardiography (HPE)* to investigate macro and micro vascular vasoreactivity [vasospastic angina, (VSA)]. This in turn can establish different ANOCA endotypes as a bedside safe cost-effective and validated modality.

#### MPSE in the assessment of ANOCA

Stress echocardiography is one of the established non-invasive diagnostic modalities to investigate patients with suspected CAD.^6^ With the advancement of ultrasound technology, transducer technology and image enhancement with AI algorithm and the implementation of ultrasonic contrast enhancement agents has led to an improvement of left ventricular regional wall thickening/motion detection and interpretation of inducible ischaemia. In addition to its diagnostic value stress echocardiography has an excellent negative and positive predictive value to predict composite MACE in short and long term.^73–75^ Moreover, beyond detecting occlusive CAD, stress echocardiography provides information of inducible ischaemia in ANOCA/INOCA patients. In addition to detecting inducible RWMA, MPSE can diagnose CMD by assessing the LAD CFVR and to prognostify patients with ANOCA. Pulse wave Doppler assessment of the mid/distal LAD CFVR as a ratio of the maximal coronary flow velocity at peak stress (maximal vasodilatation) and rest both with vasodilator and dobutamine stress echocardiography has been widely validated^15,16^ (as described above). The latest European and North American guidelines for evaluation patients ANOCA, recommend stress echocardiography with the addition of CFVR measurement (Class II level of evidence B) to improve diagnosis of CMD and for estimating risk of major adverse cardiovascular events (MACE).^6,14^ The learning curve of assessing CFVR by Doppler echocardiography is steep, however, once skills are acquired it provides a non-invasive, cost-effective, bedside test with no harmful effect of radiation for the wider chest pain population. How to assess distal LAD Doppler CFVR during stress echocardiography is provided in the supplement document (see Supplement) and has been described in a case series^76^ before, an example is shown in *Figure 4*.

#### HPE to assess vasoreactivity in ANOCA

**Figure 6:**
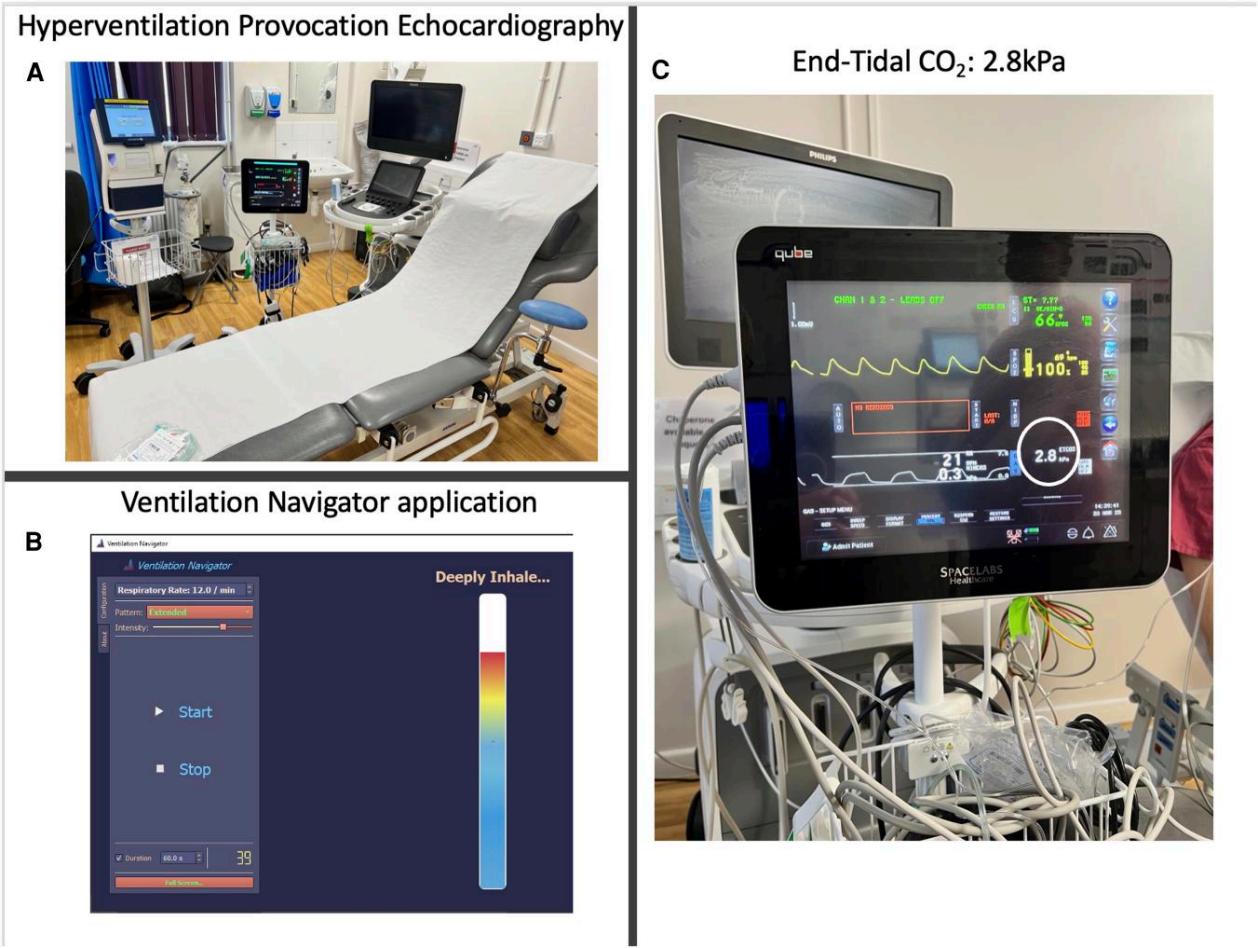
Hyperventilation provocation echocardiography setup. (*A*) Blood gas monitor device for capillary pH, and CO_2,_ Vital signs monitor for End-Tidal CO_2_, Echocardiograph for LV wall thickening assessment at rest and after 5 min of hyperventilation jointly with 12 lead ECG monitor of ST changes. (*B*) Ventilation navigator—to govern respiration rate and tidal volume (details in the Supplement). (*C*) Hyperventilating healthy proband with the capnograph showing respiration rate of 21breath per minute that resulted an end-tidal CO2 of 2.8 kPa corresponding with a capillary pH of 7.56 indicative of significant respiratory alkalosis necessary for provoking smooth muscle vasospasm of the coronary arteries if susceptible.

**Figure 7:**
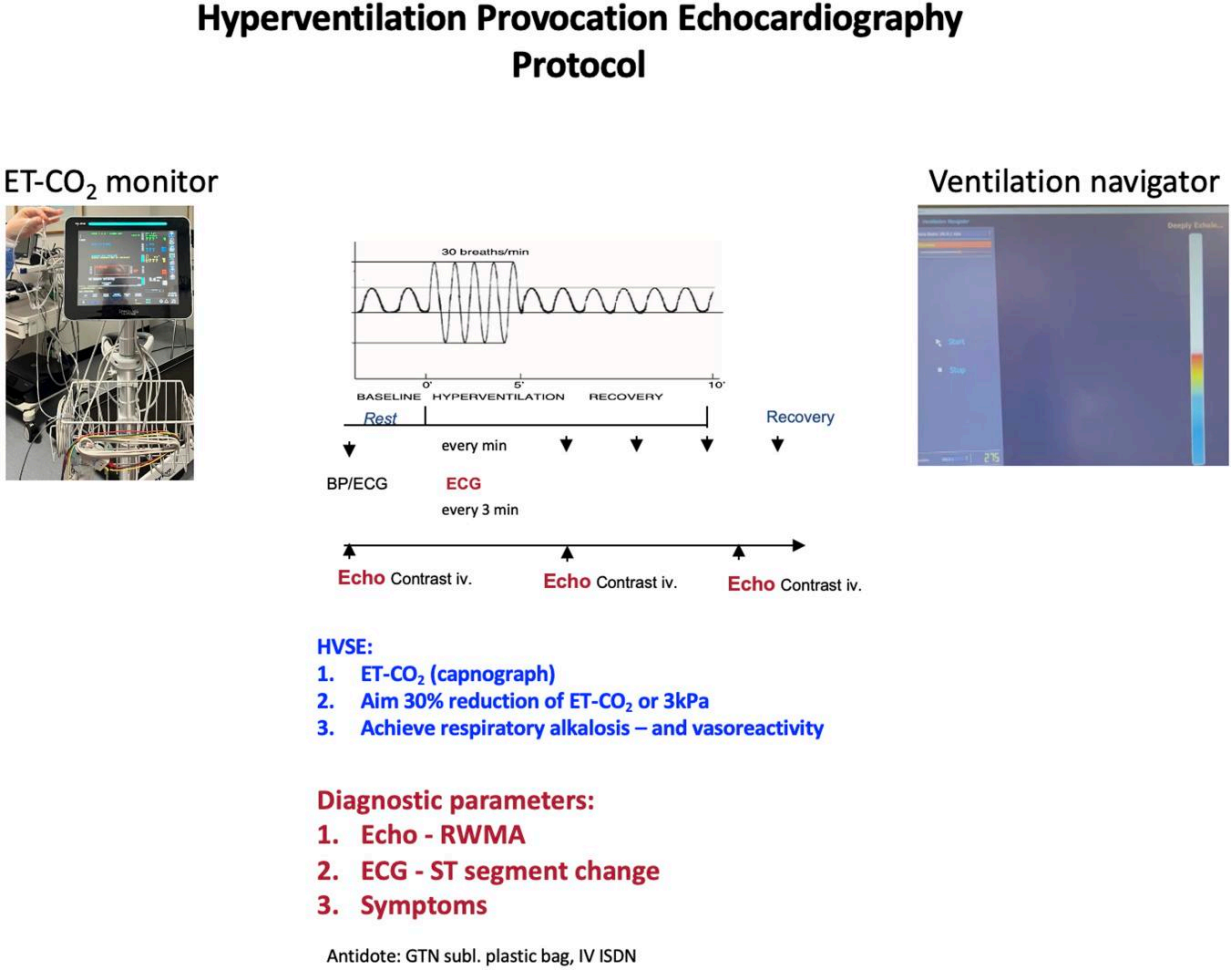
Hyperventilation provocation echocardiography protocol guided by the ventilation navigator application and controlled by ET-CO_2_. ET-CO_2_, end-tidal CO_2_; HPE, hyperventilation provocation echocardiography; RWMA, regional wall motion abnormality; ECG, electrocardiogram.

**Figure 8:**
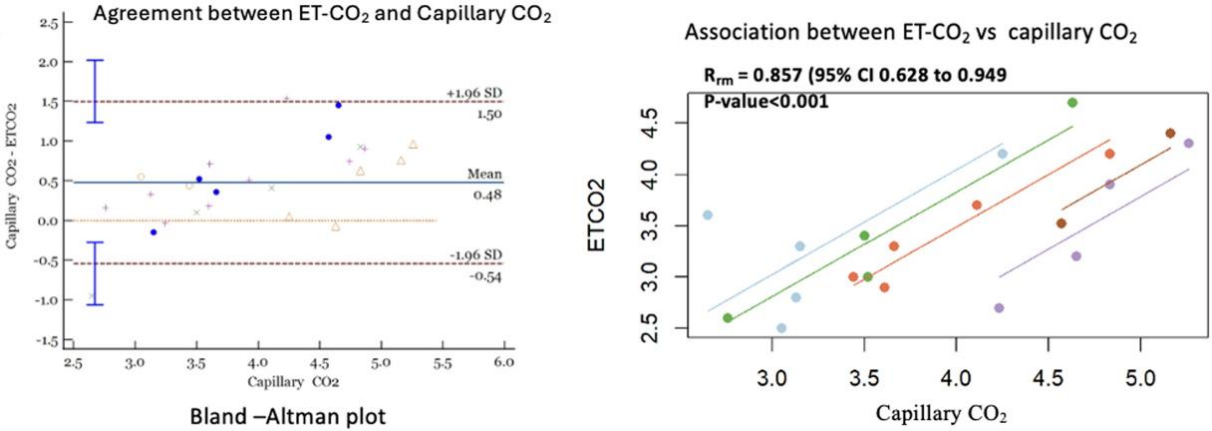
Validating controlled hyperventilation guided by the ‘ventilation navigator’ application in heathy volunteers between ET-CO_2_ and capillary CO_2_ and pH changes. Agreement and relationship between ETCO_2_ and capillary CO_2_. Bland–Altman analysis corrected for repeat measurements analysis showed a moderate, clinically relevant agreement between capillary and ETCO_2_ with an offset of 0.48 [1.50—(−0.54)] for individual data points of the probands. Linear mixed-effects modelling between ETCO_2_ vs. capillary CO_2._ (*C*) ETCO_2_ was significantly associated with capillary CO_2_ (*β* = 0737, SE = 0.115, *P* < 0.001), after accounting for repeated measures within individuals strong within individual associations for ETCO2 vs. Capillary CO_2_ as demonstrated by high Rrm: 0.857 statistical significance. Regression lines between individual are mostly parallel with no outliers in slopes, suggesting good fit of the repeated measure correlation model. (Ref:^82^)- in press in Eur Heart J-IMP 2026).

Epicardial and/or microvascular coronary vasoreactivity (VSA) is the underlying pathophysiology in 60% to 80% of ANOCA patients.^6,76,77^ There is a 21% reported overlap amongst ANOCA endotypes.^50^ In addition to the CMD, endothelial dependent vasoreactivity can be assessed with HPE with 91% diagnostic accuracy.^78–80^ We have developed a software application that is able to govern patients’ ventilation frequency and tidal volume on a visual scale (see Supplementary document and video) to achieve a respiratory alkalosis sufficient to provoke vasospasm of the pre-arterioles/arterioles or capillaries level similar to the effect of acetylcholine intracoronary injection (*Figures 6* and *7*). The proposed cellular mechanism is the hyperventilation related hypocapnia and resulting intracellular calcium overload in the smooth muscle causing vasoconstriction.^81^ The end-tidal CO_2_ (ETCO_2_), representing the partial pressure of carbon dioxide at the end of exhalation (range 4.0 to 5.7 kPa (30 −43 mmHg**)**, can be used to assess the effect of hyperventilation related hypocapnia on respiratory alkalosis (capillary blood pH >7.5). The ETCO_2_ was validated against the capillary CO_2_ and capillary pH in healthy volunteers at different respiration rates with fixed tidal volume using the Ventilation Navigator application (submitted for publication). A close agreement with a mean offset of 0.489 kPa between the ETCO_2_ and the capillary CO_2_ was found with good association of 0.857 *P* = 0.001 (*Figure 8*). Respiratory alkalosis defined as capillary pH >7.5 was achieved at different respiratory frequencies and corresponded to the ETCO_2_ of <3.0 kPa (normal range: 4.0–5.7 kPa).^82^

HPE can assesses the epicardial and microvascular coronary vasoreactivity by monitoring patients’ *symptoms*, ST segments changes on *12-lead ECG* (≥1 mm ST depression or elevation in 2 neighbouring leads), and *left ventricular wall thickening* using contrast enhanced echocardiography (*Figure 7*). It was shown that in addition to symptoms of angina and ST changes on ECG, echocardiographic assessment of inducible ischaemia has improved the diagnostic accuracy from 60% to 91% during hyperventilation.^79^ HPE can provide with a single or multi-coronary artery territory vasoreactivity assessment, albeit is not able to differentiate between epicardial and microvascular vasospasm. HPE combined with MPSE, as a fully non-invasive pathway, can also be used as a triaging tool to select patients who need further testing by IFCA.

It is important to recognize that certain comorbidities may pose significant risks during HPE, including severe pulmonary conditions such as advanced asthma, chronic obstructive pulmonary disease, and interstitial lung disease. Additionally, recent cerebrovascular events (transient ischaemic attack, stroke, intracranial haemorrhage), known cerebral artery aneurysms, or a history of sustained ventricular arrhythmia are considered contraindications for HPE in clinical practice.

## Discussion

ANOCA is prevalent amongst patients with angina with no or non-obstructive CAD.^6,14^ Exercise stress ECG has been used to make a crude assessment in ANOCA patients. Ischaemic exercise electrocardiography during stress testing identifies structural CMD in ANOCA patients with 86.3% diagnostic accuracy to identify subset of patients needing further invasive functional testing.^83^ The contemporary diagnosis of ANOCA is based on patient history, and one of the validated non-invasive tests including PET-stress test, CMR perfusion test and Doppler based stress echocardiography. These tests are assessing CFVR/MBFR as an indicator of coronary microvascular disease. None of them measures macro or microvascular vasoreactivity, albeit it accounts for 60% of ANOCA endotypes. Currently only IFCA can provide complete endotyping for ANOCA patients.^50,84,85^ This, however, is invasive with inherent risk of major complication including coronary dissection, or occlusion and even death. IFCA is not a test of choice for assessing the success of therapy. There is a need for a fully non-invasive comprehensive diagnostic pathway that is cheap, safe, with no radiation and scalable to serve our patients with suspected ANOCA to help with their diagnosis to provide targeted management and be able to follow up response to treatment.

### Proposed non-invasive diagnostic algorithm for ANOCA

**Figure 9:**
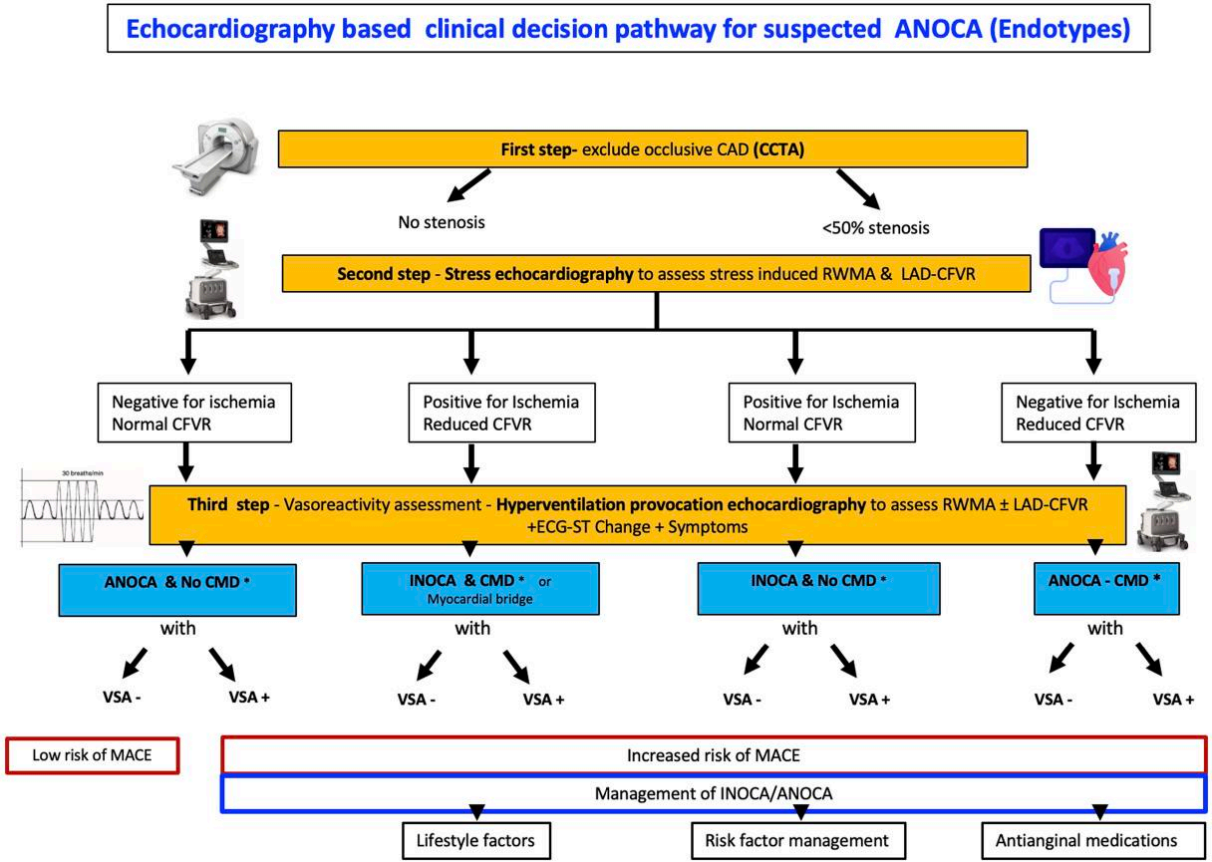
Conceptual illustration of the proposed echocardiography based clinical decision pathway for suspected ANOCA endotypes (Note the clinical utility of controlled hyperventilation to assess coronary vasoreactivity needs to be validated in prospective registries and clinical trials.).

The non-invasive echocardiography-based pathway, once occlusive CAD has been ruled out by CCTA, combining MPSE with HPE can assist in determining ANOCA endotypes based on *(i) inducible ischaemia*, *(ii) Doppler based distal LAD CFVR, and (iii) hyperventilation provocation echocardiography based vasoreactivity.* This approach can define 3 main and 4 additional endotypes with overlap features. *Figure 9* offers a conceptual overview of the diagnostic pathway for suspected ANOCA, to identify endotypes and to guide personalized management. ANOCA patients with no inducible ischaemia with normal CFVR and negative vasoreactivity testing should be considered for IFCA as a confirmatory investigation especially if symptoms and cardiovascular risk factors for ANOCA are convincing. *Table 2* provides the proposed patient specific, targeted therapy^6,86,87^ for the ANOCA endotypes determined by echocardiography based MPSE and HPE. Since the management of ANOCA patients is not an organic part of this paper we have not discussed it in detail. Nevertheless, we summarized the current state of the art recommendation of most appropriate therapies according to ANOCA endotypes in *Table 2*. *Graphical abstract* clusters the clinical conditions and/or cardiovascular risk factors associated with the individual ANOCA endotypes.

**Table 2:** Management proposal for ANOCA—endotypes (based on multiparametric stress echocardiography and hyperventilation provocation echocardiography)

ANOCA Endotypes	INOCA CMD + VSA +	INOCA CMD + VSA -	INOCA CMD - VSA +	INOCA CMD − VSA -	ANOCA CMD + VSA +	ANOCA CMD + VSA -	ANOCA CMD − VSA +	ANOCA CMD − VSA -
	Lifestyle management	Lifestyle management	Lifestyle management	Lifestyle management	Lifestyle management	Lifestyle management	Lifestyle management	Lifestyle management
	SMuRFs	SMuRFs	SMuRFs	SMuRFs	SMuRFs	SMuRFs	SMuRFs	SMuRFs
	CCB		CCB		CCB		CCB	
	Nicorandil	Nicorandil	Nicorandil		Nicorandil	Nicorandil	Nicorandil	
	Ranolazine	Ranolazine	Long-acting Nitrates	Ranolazine	Ranolazine	Ranolazine	Long-acting Nitrates	
	Ivabradine	Ivabradine		Ivabradine	Ivabradine	Ivabradine		
	Beta blockers	Beta blockers		Beta blockers	Beta blockers	Beta blockers		
		CCB		CCB		CCB		
	**NEW targets**	**NEW targets**	**NEW targets**	**NEW targets**	**NEW targets**	**NEW targets**	**NEW targets**	
	Endothelin antagonist	Endothelin antagonist	Endothelin antagonist	Endothelin antagonist	Endothelin antagonist	Endothelin antagonist	Endothelin antagonist	
	Phosphodiesterase inhibitor	Phosphodiesterase inhibitor	Phosphodiesterase inhibitor	Phosphodiesterase inhibitor	Phosphodiesterase inhibitor	Phosphodiesterase inhibitor	Phosphodiesterase inhibitor	
	Pho Kinase inhibitor	Pho Kinase inhibitor	Pho Kinase inhibitor	Pho Kinase inhibitor	Pho Kinase inhibitor	Pho Kinase inhibitor	Pho Kinase inhibitor	
	Cell and gene therapy	Cell and gene therapy	Cell and gene therapy	Cell and gene therapy	Cell and gene therapy	Cell and gene therapy	Cell and gene therapy	
	Fatty acid oxidation inhibitors	Fatty acid oxidation inhibitors	Fatty acid oxidation inhibitors	Fatty acid oxidation inhibitors	Fatty acid oxidation inhibitors	Fatty acid oxidation inhibitors	Fatty acid oxidation inhibitors	
	Coronary sinus reducer	Coronary sinus reducer	Coronary sinus reducer	Coronary sinus reducer	Coronary sinus reducer	Coronary sinus reducer	Coronary sinus reducer	
				***Consider for IFCA***				***Consider for IFCA***

SMuRFs, Standard Modifiable Risk Factors (Hypertension, Diabetes, Dyslipidaemias, and Smoking); INOCA, ischaemia with non-occlusive coronary arteries (i.e. inducible Regional Wall Motion Abnormality during stress echocardiography); ANOCA, angina with non-occlusive coronary arteries (i.e. no-inducible Regional Wall Motion Abnormality during stress echocardiography); CMD, coronary microvascular disease; VSA, vasospastic angina; CCB, calcium channel blocker; IFCA, invasive functional coronary angiography (ref:^6,86,87^).

## Conclusion

The echocardiography based MPSE in combination with HPE can diagnose CMD and vasoreactivity based on ischaemia, CFVR and endothelium dependent coronary vasospasm in ANOCA patients. Seven distinct endotypes of ANOCA can be identified to be able to deliver targeted therapy for symptomatic control, to avoid recurrent hospital admissions and reduce healthcare costs. The proposed echocardiography based non-invasive pathway, as a scalable, bedside, cost-effective test makes it an attractive proposition as a future diagnostic modality of ANOCA endotypes, to risk stratify and to monitor treatment effect. In case of uncertainties in diagnostic accuracies, it can also serve as a triaging tool for patients who would benefit from downstream testing by IFCA. The clinical utility of the proposed pathway should be tested in prospective registries and clinical trials.

## Supplementary Material

qyag129_Supplementary_Data

## Data Availability

No new data were generated or analysed in support of this research.

## Lead author biography

Professor Attila Kardos is a consultant cardiologist in Milton Keynes University Hospital, where he is also the Associate Director of Research and the Director of the Translational Cardiovascular Research Group that is affiliated to University of Buckingham. He has a Honorary Chair at the University of Buckingham as Professor of Cardiovascular Medicine, and holds a Visiting Professorial position at both Cranfield and Open Universities.

He is an Honorary Senior Lecturer to the Division of Cardiovascular Medicine at the Radcliffe Department of Medicine, University of Oxford. He is the director of the Multimodality Cardiovascular Imaging Services in Milton Keynes. He has over 25 years of research experience and 32 years of clinical experience of which the last 23 years as a consultant.

Professor Kardos is a chief investigator in several research trials and projects. His research interest expands to cardiovascular imaging modalities (CCTA and stress echocardiography) pathophysiology of stable and acute coronary syndromes in particular ANOCA, AI algorithm in cardiovascular diagnostics, and predictive modelling, AI and Robotics in cardiovascular imaging.

He has published over 125 peer-reviewed journal articles, H-index: 24; He has been a Invited as well as Keynote speaker at National and International Conferences >120. He has supervised 5 MSc, DPhil/PhD/MD students Professor Kardos is a Fellow of the RCP and the European Society of Cardiology and a member of several International Societies such as EACVI, SCCT, BCS, AHA, ACC, ASE etc. Associate Editor of Frontiers in Cardiovascular Medicine, Journal of Clinical Medicine, Cardiovascular Endocrinology & Metabolism, and reviewer to several Journals, e.g. The Lancet, Open Heart, Open BMJ, JMIR, Frontiers in Cardiovascular Medicine.
