# Supplementary material for: A proposal of an echocardiography-based pathway for the diagnosis and management of angina with no obstructive coronary arteries: a state-of-the-art review: Advancing Targeted Therapy and Prognosis Through Endotype Identification
Source: Eur Heart J Imaging Methods Pract. 2026 Jul 22;4(3):qyag129. doi: 10.1093/ehjimp/qyag129 (PMC13449611; doi:10.1093/ehjimp/qyag129)
Supplement: Supplementary file 1 [file qyag129_supplementary_data.zip › Supplementary document.docx]

Supplementary documents.

**Review**

**State of the Art Diagnosis of ANOCA – A Proposal of a Comprehensive Non-Invasive Echocardiography-Based Pathway for the Diagnosis and Management of Angina with No Obstructive Coronary Arteries**

Advancing Targeted Therapy and Prognosis Through Endotype Identification

Attila Kardos MD., PhD., FRCP. FESC ^1,2^ Benjamin Bussmann BA BMBCH ^3,4.^ Maria George ^5^ Giovanni Luigi De Maria MD, PhD, FESC ^3,6^ Nicola Gaibazzi, MD., PhD ^7^

**Affiliations:**

1 Department of Cardiology, Translational Cardiovascular Research Group, Milton Keynes University Hospital NHS Foundation Trust, Milton Keynes, United Kingdom

2 Faculty of Medicine and Health Sciences, University of Buckingham, Buckingham, United Kingdom

3 Department of Cardiology, Oxford University Hospital, Oxford, United Kingdom

4 Department of Physiology Anatomy and Genetics, University of Oxford

5 INOCA International, Manchester, United Kingdom

6 AMIIC Catheterization Laboratory, University of Oxford, Oxford Heart Centre – Oxford University Hospital

7 Cardiology Department, Parma University Hospital, Italy

**Corresponding Author:**

Prof. Attila Kardos, MD, FRCP, PhD, FESC,

Department of Cardiology, Translational Cardiovascular Research Group, Milton Keynes University Hospital NHS Foundation Trust, Milton Keynes, United Kingdom, 8H Standing Way, Eaglestone, Milton Keynes, MK6 5LD UK.

Email: [attila.kardos@cardiov.ox.ac.uk](mailto:attila.kardos@cardiov.ox.ac.uk)

**Disclosures**: The authors have nothing to disclose.

**Funding:** No funding was received for this work.

***Word count: 3544***

***Table: 2***

***Figure: 10***

***References: 84***

**Supplementary Document**

A. **Technical description of LAD coronary flow velocity reserve (CFVR) measurement by Doppler echocardiography**:

To measure CFV coronary arteries must first be visualized by colour Doppler imaging. Distal segments are preferred because in case of severe stenosis the post-stenotic flow decreases, compared to the pre-stenotic flow. LAD is more readily available for visualization than other coronary arteries (successful visualization rate varies between 35-96% for distal LAD, 33-91% for PDA, 0-33% for distal LCX). In terms of dobutamine stress echocardiography due to the increased heart rate the diastolic time shortens, and the CFV measurement become more challenging in all coronary arteries with more success in the LAD. However, if CFV of PDA and LCX is of interest vasodilator stress test is more appropriate (Boshchenko et al. 2011). Nevertheless, in patients with INOCA both vasoreactivity or CMD is effecting all three coronary artery circulation, hence to interrogate CFV in LAD deems sufficient.

For CFV assessment we use frequencies 3.7-4MHz for LAD and 3.1-3.3 for PD and OM at baseline and 3.3 MHz for LAD at peak dobutamine with harmonic. Velocity scale is set at 21cm/sec at baseline and 50cm/sec at peak for colour and pulse wave Doppler. If we cannot detect LAD at baseline, we reduce the scale further to 10cm/sec. We are using Sonovue as UEA administered in bolus doses of 0.2-0.5ml before each stage of the SE for left ventricle opacification before image capture. UEA augments Doppler signals can also amplify noise and makes CFV measurements more difficult. To overcome this problem the CFV assessment at peak is performed before contrast administration. Nota-bene: lowering colour and PW gain can help. The most common pitfall would be to compare velocities measured with different beam angle. We do not use Doppler angle correction and aim to position the probe so that the ultrasound beam is parallel to the vessel as much as possible.

In our practice the visualization rate of the LAD at rest is 82,3%. Those with resting LAD CFV 84% will CFV measured at peak for CFVR calculation during dobutamine stress test, and 97% will have CFVR calculated during dipyridamole stress test.

In our experience since the superficial location of the LAD near the chest wall, a large BMI isn't a problem in visualizing LAD; in fact, the more "horizontal" position of the heart due to diaphragm position the more helpful to visualise the LAD. COPD is more challenging co-morbid condition; a) inflated lungs limit the echo window and b) these patients are not being able to hold their breath for coronary flow acquisition. Since inspiratory or expiratory movements have an impact on CFV we are aiming to acquire CFV measurements during breath hold.

**Reference**

Boshchenko A, Vrublevsky A, Karpov R. Transthoracic Echocardiography in the Assessment of Coronary Arteries. Coronary Angiography - Advances in Non-invasive Imaging Approach for Evaluation of Coronary Artery Disease. Prof. Baskot Branislav (Ed.), ISBN: 978-953-307-675-1, InTech. 2011;22-60.

B. **Ventilation Navigator software application**

The Ventilation Navigator software serves as an advanced platform for respiratory guidance and control, incorporating a comprehensive range of features such as adjustable breathing rate, and tidal volume, a session timer for automatic cessation, and customisable breathing pattern settings. Its graphical user interface is purposefully designed to deliver clear and engaging visual cues to the patient, facilitating precise regulation of respiratory movements whilst minimising distractions and cognitive demand. Developed in C++ and utilising high-precision timers, the software ensures optimal accuracy and reliability for clinical applications.

**Supplementary Video capture:**

Ventilation Navigator – to govern respiration rate and tidal volume (details in the methods).

The Ventilation Navigator application was developed as an advanced platform for respiratory guidance with adjustable respiration rate, and tidal volume. The session timer for automatic cessation, of the task. Its graphical user interface is designed to deliver clear and engaging visual cues to the patient.
